# Non-Viral Nanovectors Based on Cyclodextrins for siRNA Delivery: An Update to Current Technologies

**DOI:** 10.3390/pharmaceutics18020265

**Published:** 2026-02-21

**Authors:** Ilaria Chiarugi, Francesca Maestrelli, Giulia Piomboni, Sandra Ristori, Anna Rita Bilia

**Affiliations:** Department of Chemistry “Ugo Schiff” (DICUS), University of Florence, Via Ugo Schiff 6, 50019 Florence, FI, Italy; ilaria.chiarugi@unifi.it (I.C.); giulia.piomboni@edu.unifi.it (G.P.); sandra.ristori@unifi.it (S.R.); ar.bilia@unifi.it (A.R.B.)

**Keywords:** cyclodextrins, modified-CD, siRNA, gene delivery, nanovectors

## Abstract

Gene delivery/administration and, in particular, small interfering RNA (siRNA) delivery represent a therapeutic challenge, though very effective carriers have yet to be identified. Cyclodextrins (CDs) are cyclic oligosaccharides with unique host–guest inclusion capabilities, widely recognized in the pharmaceutical field for their ability to enhance drug solubility and bioavailability. Their excellent biocompatibility and chemical versatility make them powerful building blocks for the design of supramolecular nanovectors (NVs). Thanks to their facility of functionalization, CDs are highly versatile and have found numerous applications across various fields. In this context, CD-based NVs are currently explored as non-viral agents to transport and release siRNA. Recent studies suggest that self-assembled NVs based on CDs can improve the transfection and safety of siRNA delivery. This review provides a comprehensive overview of the most recent advances in the design of NVs based on CDs and their use for siRNA delivery, discussing the role played by structural differences and chemical functionalization in the context of encapsulation and release.

## 1. Introduction

Gene therapy is a promising medical approach to achieving the accurate and personalized cure for diverse severe pathologies. The discovery of RNA interference (RNAi) by Fire and Mello in 1998 laid the bases for a new gene therapy approach, recognized with the Nobel Prize in Physiology or Medicine in 2006 [1]. RNAi is a post-transcriptional gene silencing RNA, which acts against endogenous parasitic and exogenous pathogenic nucleic acids, with consequent regulation of the expression of protein-coding genes. RNAi is mediated by short non-coding (approximately 22 nucleotides) RNA, the small interfering RNA (siRNA), and the microRNA (miRNA). From the perspective of therapeutic development, siRNA is very interesting due to its specificity, which minimizes off-target effects, and since 2001, synthetic siRNAs have been developed, providing the foundation for therapeutic applications [2,3], as reflected by the high number of clinical trials rapidly developed in recent years [4] as well as by the approved siRNA medicines on the market of the USA and Europe [5,6]. CDs are cyclic oligosaccharides composed of α-(1 → 4)-linked glucopyranose units, most commonly naturally existing as α-,β-, and γ-CDs containing six, seven, and eight glucose units, respectively. Due to their unique toroidal structure with a hydrophilic outer surface and a hydrophobic inner cavity, CDs can form host–guest inclusion complexes with a wide variety of molecules, ranging from small organic compounds to macromolecular assemblies. This supramolecular capability has made CDs valuable tools in fields such as drug delivery, catalysis, food technology, and environmental remediation [7]. CDs are EMA- and FDA-approved excipients with low toxicity and immunogenicity, but concerning the cationic derivatives, still, most of the information about their toxicity derives from in vitro studies, and there is a lack of knowledge on their interaction within the body, as well as on the mechanism of elimination of these carriers. Indeed, studies on the molecular mechanism of interaction between cationic CDs and biological components are still in their infancy [8,9]. The in vitro models, including specific cell lines or bacterial strains, greatly limit their translation to humans or animals [10]. Only in vivo studies can provide insights concerning their safety profiles, biocompatibility, and biodistribution [11]. β-CDs are the most frequently employed derivatives in pharmaceutical research because they offer an optimal balance between cavity dimensions and stability, providing robust support for siRNA binding and versatile chemical derivatization. In pharmaceutical sciences, CDs have mainly been employed to enhance solubility, stability, and bioavailability of poorly soluble drugs and to control release profiles while reducing drug toxicity. Their high biocompatibility, low immunogenicity, and great chemical versatility have further expanded their application scope from small-molecule encapsulation to the design of advanced nanostructured materials for biomedical purposes. Chemical modification of hydroxyl groups on CD rims allows for the introduction of functional moieties (cationic, amphiphilic, targeting ligands, or stimuli-responsive groups), thereby tailoring their physicochemical and biological properties [12,13,14]. In recent years, these features have been exploited in the development of CD-based supramolecular systems for gene delivery, including DNA and siRNA vectors. CDs can serve as non-viral carriers that condense, protect, and deliver nucleic acids to target cells while minimizing cytotoxicity and immunogenic responses. A first review on the use of CDs for siRNA delivery was written by Chaturvedi et al. about 15 years ago [15]. More recently, the review by Mousazadeh et al. has evidenced the potential of cyclodextrins as effective non-viral siRNA delivery systems for cancer gene therapy [16], suggesting that modification of CDs can improve transfection and safety of delivery systems. In this framework, the review by Hu et al. (2023) further highlighted the dual diagnostic and therapeutic potential of CD-based systems [17]. Specifically, it discussed how functionalized CDs can serve not only as siRNA carriers but also as multifunctional platforms for imaging, controlled release, and targeted cancer therapy, bridging the gap between nanomedicine and theragnostics. Taken together, these studies underscore the versatility of CD architectures as robust and adaptable frameworks for next-generation gene delivery technologies. The primary objective of the present review is to provide an updated overview of the most recent advances in modified CD-based NVs for siRNA delivery, with a focus on structural design and functionalization strategies. The main thermodynamic/kinetic aspects that influence complex formation and intracellular release are also discussed. Accordingly, this review is structured into three main sections. The review firstly focuses on the structural design and chemical functionalization of cyclodextrin-based nanovectors, describing the most relevant strategies employed to obtain cationic, amphiphilic, and stimuli-responsive derivatives. Then, the physicochemical and biological performance of these systems, with particular emphasis on siRNA complexation, protection from enzymatic degradation, cellular uptake, and gene silencing efficiency, have been deeply discussed. Finally, the review addresses critical challenges and future perspectives, including structure–function relationships, intracellular release mechanisms, safety considerations, and translational limitations of cyclodextrin-based siRNA delivery systems

## 2. Challenge and Strategies in siRNA Delivery

Despite the ideally promising scenarios for siRNA therapeutic applications, several intracellular and extracellular barriers limit siRNA clinical use. These are mainly inadequate stability and a limited pharmacokinetic profile, together with the possible stimulation of unwanted side effects. Indeed, RNases and phosphatases can degrade the phosphodiester bond, while enzymes found in serum and tissues can prevent its accumulation in the target tissue. A major limitation of naked siRNA is its high susceptibility to enzymatic degradation by serum nucleases, which rapidly cleave the phosphodiester backbone and severely limit circulation half-life. Naked siRNA-based drugs can only represent an effective strategy for local delivery, as in the case of eye treatments. Additionally, the size of siRNAs (about 7–8 nm in length and 2–3 nm in diameter), their hydrophilicity, and their polyanionic nature present some difficulties in penetrating the membrane lipid bilayer. Therefore, siRNA can be easily cleared by glomeruli and excreted within a time lapse from several minutes to one hour [18]. A further intracellular difficult step is the endosomal escape, and innate immune activation can occur when naked siRNA is used. Naked siRNAs leaving the bloodstream are accumulated in the bladder and quickly (a few minutes to half an hour) excreted from the body, which prevents their accumulation in the target tissues or cells. Various chemical modifications have been proposed to obtain clinically efficient medicines, together with the use of cationic cell-penetrating peptides. Additionally, N-acetylgalactosamine-conjugated siRNA has been largely used to enhance metabolic stability, and nowadays many drugs such as givosiran, lumasiran, inclisiran, vutrisiran, and nedosiran have been approved by the FDA for this purpose. However, this strategy is very successful for liver targeting by the asialoglycoprotein receptor [19,20].

Encapsulation or complexation of siRNA within nanovectors is therefore essential [21,22]. The use of drug delivery systems, in particular NVs, is an exciting prospect to avoid quick renal clearance and, more importantly, to obtain selective targeting of cells and tissues. Due to many limitations of virus-based vectors, drug delivery systems using lipids and cationic polymers, which provide the electrostatic binding of negatively charged siRNA, are suitable vectors [23]. Lipid nanoparticle (LNP) transport of siRNA has become one of the most advanced approaches in the field of RNAi therapeutics [18]. The first approved drug employing this technology is patisiran (ONPATTRO^®^), recommended for the treatment of hereditary transthyretin amyloidosis (hATTR) [24]. Since then, several candidates have entered clinical development, including BMS-986263 (Bristol-Myers Squibb), an LNP formulation carrying siRNA targeting HSP47 for the treatment of hepatic fibrosis [25]. Another type of NVs is represented by polymeric nanoparticles (PLGA, PEG, chitosan, PEI, and other cationic or ionizable polymers). They have also been explored as versatile siRNA delivery systems, offering tunable design parameters, controlled release, and robust nucleic acid protection, despite challenges such as potential cytotoxicity of certain cationic polymers and generally lower clinical maturity compared to LNPs [26,27].

Cationic micelles have also been used for the transport of small oligonucleotides and, in particular, siRNA [28,29]. Due to the possibility of monomer exchange, micelles present relatively flexible structures that can be squeezed or loosened to allow cargo loading and delivery. Moreover, their simple architecture can be modeled by computational methods, and the obtained information can be transferred to more complex systems based on other soft matter aggregates.

CDs are natural cyclic oligosaccharides derived from starch, characterized by a ring structure with hydrophilic primary and secondary sides and a hydrophobic cavity [30]. Due to the presence of hydroxyl groups, CDs can be easily functionalized to impart a positive charge to the molecule [31]. More generally, chemical functionalization can produce an unlimited number and variety of CD derivatives, including cationic, amphiphilic, and PEGylated compounds. In particular, the electrostatic interactions between nucleic acid and cationic CDs provide additional self-assembly mechanisms.

As recently reported by Nazli and coworkers [32], the positively charged vectors can strongly interact with negative charges, promoting robust cellular interactions, improving permeability and cell uptake of siRNA, and increasing its activity. In addition, the amine groups of cyclodextrins facilitate the so-called “proton sponge effect”, causing an increased flux of water with possible endosomal rupture and consequent release of siRNA in the cytoplasm, improving the biological effects as well. Furthermore, these effects can lead to cell toxicity effects because the strong electrostatic interactions of nanovectors can lead to an alteration of the cell membrane with consequent cell damage. An optimal balance between maximizing effective siRNA delivery and minimizing toxicity is critical for a successful therapy. From the available studies, it is clear that these nanovectors guaranteed site-specific drug release, maintaining high cell viability as compared to commercially available vectors such as PEI25 kDa and lipofectamine 2000. As recently reported by Pirvu et al. [33], cationic CD derivatives form nanoparticles that protect siRNA, promote endosomal escape, and target delivery while presenting low immunogenicity. Concerning their behavior in aqueous solution, it has been clearly established [34,35] that, like all other amphiphilic molecules, CDs undergo cooperative self-assembly into equilibrium structures that are in dynamic exchange with the monomers, according to the classical pseudophase model [36,37]. The large number of OH groups in the sugar ring, by allowing strong hydrogen bonds and hydration forces, plays a key role in this process, though, especially for functionalized CDs, hydrophobic interactions should also be taken into account. Moreover, if ionic groups are present in the molecule, the electrostatic contribution brings a further term in the free energy of aggregation, thus making the whole process very complex to model in quantitative terms [38,39]. Such complexity is mirrored by the different names and attributes given to the spontaneous aggregates that CDs form in water solution (transient clusters, nanoparticles, microparticles) [40,41,42,43,44], though all the standard techniques for establishing the onset of aggregates, including scattering, microscopy, and spectroscopy [45,46,47], agree on the presence of entities other than the monomers above a critical concentration value.

Beyond protection, an effective siRNA delivery system must also balance binding strength and reversibility, in that excessive stabilization can hinder intracellular release and reduce the gene silencing efficiency. This trade-off represents a central design challenge for non-viral nanocarriers [48].

Taken together, CD-based non-viral platforms illustrate how a diverse range of nanocarriers can offer complementary solutions to the challenges of siRNA transport, protection, and cellular uptake. Each platform brings distinct structural features, mechanisms of interaction with nucleic acids, and opportunities for tissue targeting, while also presenting specific limitations related to stability, biocompatibility, manufacturing, or clinical translation. To provide a consolidated overview, Table 1 summarizes the key attributes discussed in this section, highlighting the main advantages, drawbacks, and references cited in this article.

CD NVs are particularly promising, and significant research activity is currently underway to refine their properties. Despite this progress, substantial work is still required to fully exploit their potential and translate these systems into clinically validated siRNA delivery platforms.

Haley et al. (2020) have provided a comprehensive overview of CDs as modular carriers in drug and gene delivery systems [49]. Their review illustrated how the chemical versatility of CDs allows for the complexation and protection of DNA and RNA, reduction in immunogenicity, and facilitated interactions with cell membranes. It also highlighted the ability of CDs to serve as platforms for combined systems—including chemotherapeutics, RNAi, and peptides—with potential applications in personalized therapies. However, this analysis was focused on studies up to 2020, leaving room for an update that includes more recent evidence of self-assembling and targeted systems for siRNA delivery. Table 2 provides a concise overview of the CD-based NVs reported in the literature.

## 3. NVs Based on Modified CD for siRNA Delivery

CD-based NVs have gained increasing attention due to their biocompatibility, their ability to form stable complexes with siRNA, and their potential for targeted functionalization. Moreover, it has been reported that the combination of CDs with cationic polymers can promote cell penetration [32]. Selective chemical functionalization of cyclodextrin rims further enables the fine-tuning of surface charge through the introduction of cationic moieties, such as polyethylenimine or amino acid residues, ensuring efficient siRNA condensation and protection. In addition, the conjugation of targeting ligands (e.g., folic acid or anisamide) can promote site-specific cellular uptake via receptor-mediated endocytosis, while subsequent endosomal escape is facilitated by proton sponge effects or by the inherent membrane-disruptive properties of the cyclodextrin-based nanovectors.

**Figure 1:**
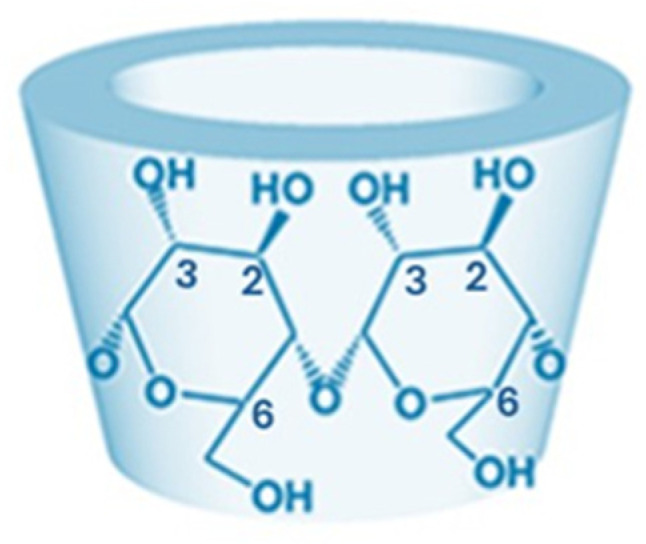
Modifiable groups of β-CDs.

The main portions of CD that are modified are the hydroxyl groups at positions 2 and 3, and the CH_2_OH group at position 6 (Figure 1).

**Figure 2:**
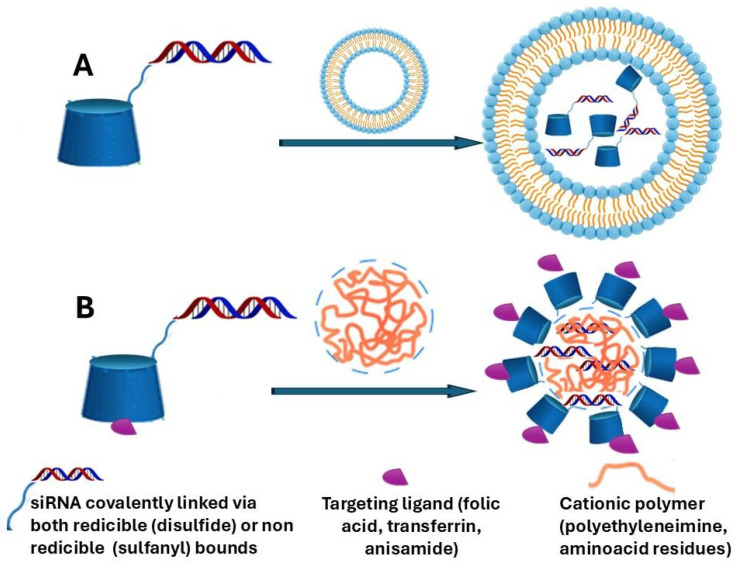
Conceptual representation of NVs developed by Malhotra et al. (2018) [51]. Panel (**A**) represents the lipofectamine-based system incorporating CD and siRNA, while Panel (**B**) illustrates the amphiphilic CD system complexed with siRNA.

Therefore, it appears that modification of the native CD structure is highly recommended for internalization. As an example, amphiphilic cationic CDs have been studied for siRNA release, showing success in mediating gene silencing both in vitro and in vivo. Malhotra et al. present the first example of CD-siRNA conjugates for gene silencing applications [51]. In this study, β-CD was covalently attached to the sense strand of siRNAs via both reducible (disulfide) and non-reducible (sulfanyl) linkers. The conjugates maintained gene silencing efficacy comparable to unconjugated siRNAs when delivered via polycationic lipids such as Lipofectamine 2000 [52] and amphiphilic cationic CDs (Figure 2).

**Figure 3:**
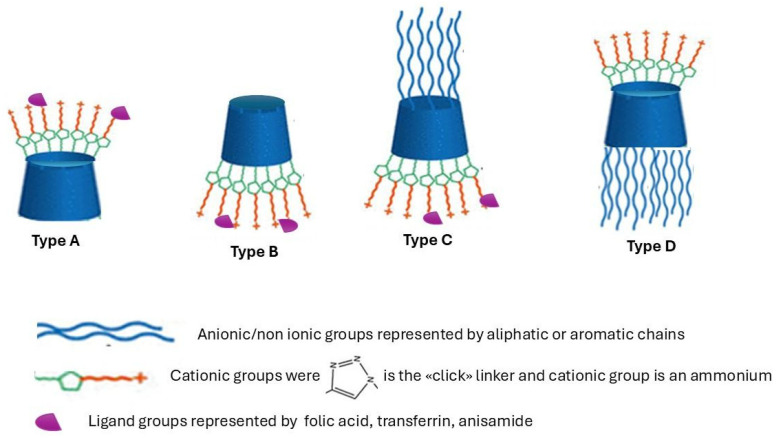
Functionalization of CD derivatives.

CD NVs are also able to overcome physiological barriers such as the blood–brain barrier (BBB) and deliver siRNA to malignant or genetically affected tissues, underscoring their promise for the treatment of neurodegenerative diseases [53]. The system utilized amine-functionalized β-CDs, which facilitated electrostatic complexation with siRNA and promoted endosomal escape. These nanoparticles demonstrated efficient transcytosis across the BBB, along with significant gene -silencing effects in neuronal cells. Different approaches can be used to improve the interactions between CDs and siRNA, and in addition, targeting ligands can be added to improve siRNA transfection. Specifically, the PEGylation process can be used as passive targeting and as a means for prolonging systemic circulation time. Following this idea, adamantane–transferrin and adamantane-–PEG derivatives of CDs have been produced, enhancing the performance of CD-based platforms by double functionalization with one targeting moiety [54]. In Figure 3, the main types of functionalization of CDs explored in this review are reported. Different structures of cationic CDs are obtained by conjugation with a cationic polymer, which can be linked to the smaller opening of the toroid (primary rim) corresponding to the C6-OH primary hydroxyls (type A) and the larger opening (secondary rim) to the C2-OH and/or C3-OH secondary hydroxyls (type B). Additionally, the modified cyclodextrins can be substituted with anionic or non-ionic polymers in the other rims (type C and type D).

Moreover, amphiphilic CDs, which possess both hydrophilic and hydrophobic domains due to substitution with aliphatic or aromatic chains, can undergo hydrophobic-driven self-assembly into core–shell structures or micelle-like NVs. CD-based siRNA nanovectors are typically formed by self-assembly under mild aqueous conditions. This behavior is particularly advantageous for siRNA delivery, as it enables formulation under physiological conditions without harsh organic solvents or high-energy processes while allowing for size control, targeting moiety integration, and siRNA protection in a single-step process. In such configurations, siRNA is either electrostatically adsorbed to the particle surface or co-condensed within the hydrophilic shell. These amphiphilic assemblies improve NVs’ stability at physiological conditions and promote membrane fusion or cellular endocytosis. This process may involve the complexation of cationic CDs with the anionic siRNA. In this case, the encapsulating CD superstructure can preserve the native siRNA conformation through a vast network of hydrogen bonds between the positively charged side arms of the c-CD and the negatively charged siRNA skeleton. Additionally, CDs can participate in supramolecular self-assembly via host–guest interactions, such as the inclusion of hydrophobic guest molecules (e.g., adamantane and, cholesterol) within the CD cavity. This approach has been widely exploited to construct modular NVs by linking targeting ligands (e.g., transferrin, folic acid), PEG, or additional stabilizing elements via guest moieties, resulting in highly customizable delivery platforms [55]. In systems where CDs are functionalized with cationic side chains (e.g., amino, guanidinium, or polyethyleneimine-like moieties), electrostatic complexation with the negatively charged phosphate backbone of siRNA can drive spontaneous nano-condensation into stable nanoparticles. The resulting complexes can preserve the conformational integrity of siRNA, protecting it from serum nucleases and enhancing cellular uptake.

Seripracharat et al. (2022) describe the development of a supramolecular siRNA delivery system based on the host–guest assembly between cationic β CD derivatives (cCDs) and an adamantane-functionalized poly(vinyl alcohol)-poly(ethylene glycol) (Ad PVA PEG) polymer [56]. Three distinct amino-substituted cCDs—bearing putrescine, spermidine, or spermine moieties—were synthesized and confirmed via ^1^H NMR and mass spectrometry. These cCDs spontaneously form spherical nanoparticles with Ad PVA PEG and siRNA, as shown by ^1^H NMR and SEM, with particle sizes below 300 nm and a negative zeta potential at physiological pH. Gel electrophoresis demonstrated efficient siRNA loading (≈90%), while DLS analysis confirmed stable complexation. In vitro assays in A549 cells indicated effective GFP gene silencing, comparable to Lipofectamine™ 2000, with minimal cytotoxicity. Kont et al. 2022 report a novel strategy for siRNA delivery using co-formulated amphiphilic cationic and anionic β-CDs to treat acute myeloid leukemia (AML) [57]. A newly synthesized anionic amphiphilic CD was blended with a cationic CD complexed with siRNA targeting the epigenetic regulator KAT2a. The resulting nanoparticles displayed reduced surface charge (from +34 mV to +24 mV) and improved polydispersity, without compromising particle size or siRNA uptake (~60% in HL-60 AML cells). Despite a slightly slower endosomal escape for the co-formulated NVs, both formulations achieved comparable gene silencing (~21–29% KAT2a knockdown). These findings highlight the potential of charge-balanced CD nanocarriers to minimize toxicity while maintaining therapeutic efficacy for non-viral gene delivery in hematological malignancies. Sun et al. (2023) report the development of sialic acid-functionalized CD-based nanoparticles for targeted delivery of CSF-1R siRNA to tumor-associated macrophages (TAMs) in prostate cancer [58]. These findings demonstrate promising in vitro gene silencing efficacy with low cytotoxicity; however, further in vivo validation is required to assess biodistribution and therapeutic relevance [56,57,58]. The sialic acid ligand enables selective binding to Siglec-1 (CD169), highly expressed on M2-like TAMs, facilitating efficient uptake and significant CSF-1R gene silencing (42–58% knockdown vs. 19–39% for non-targeted controls; *p* < 0.01). The suppression of CSF-1R expression induces macrophage repolarization from the immunosuppressive M2 phenotype to pro-inflammatory M1, demonstrated by increased CD86+/CD68+ populations (~72%) and reduced CD206+/CD68+ (~25%). In co-culture models with prostate cancer cell lines (PC-3, TRAMP-C1), this macrophage reprogramming leads to enhanced cancer cell apoptosis (49–69% vs. 38–44%; *p* < 0.01). Hao et al. (2024) describe a hybrid nanoparticle system combining AS1411 aptamer–PD L1 siRNA chimera with glutamine-modified carboxymethyl-β-CD (Gln CM-β CD) and polyethylenimine/doxorubicin for combinatorial chemo-immunotherapy against lung squamous cell carcinoma [59]. Importantly, these results were further supported by in vivo studies, confirming therapeutic efficacy and immune modulation in relevant tumor models [59]. The AS1411 aptamer directs selective binding and internalization into NSCLC cells, achieving effective PD L1 silencing and stimulation of T cell and CD8^+^ cytotoxic responses. SEM imaging revealed conical nanoparticles (~250–500 nm), while glutamine modification enhanced doxorubicin uptake and apoptotic induction in tumor cells. In vivo studies demonstrated superior tumor inhibition (reduced volume and Ki 67 index, increased apoptosis) and elevated intra-tumoral T cell infiltration (1.34- to 1.41-fold increase in CD8^+^ T cells), with reduced systemic toxicity compared to aptamer-only or chemotherapy-alone controls. Although no CD NV–based siRNA therapeutics have reached market approval so far, they represent an important area of preclinical research, with promising applications in oncology, neurology, and inflammatory diseases, underscoring their potential role in the future of non-viral gene therapy. Some formulations based on CD NVs are currently in clinical trials, such as, for example, CALAA-01, a self-assembling CD NV (CALANDO) functionalized with transferrin for tumor targeting. This system was designed for delivering siRNA against the RRM2 gene. The designed clinical trial product is a combination of siRNA (USP # 7427605, 23 September 2008) and RONDEL (United States Patent (USP) # 7807198, 5 October 2010). The Phase I clinical trial has provided the first evidence of siRNA-mediated gene silencing in human tissues following systemic administration and represents a significant milestone in RNAi therapeutics [60]. Another example is siG12D-LODER, a biodegradable intratumoral implant based on CD NVs that has advanced to Phase II for the treatment of pancreatic cancer, demonstrating sustained siRNA release directly within the tumor microenvironment [61].

### Stimuli Responsive and Thermodynamics in CD-Mediated Gene Delivery

Following cellular internalization, siRNA-loaded nanovectors are typically entrapped within endosomal compartments, where insufficient release into the cytosol remains a major bottleneck. Efficient endosomal escape and controlled intracellular disassembly are therefore crucial to enable siRNA access to the RNA-induced silencing complex (RISC) [62,63].

NVs CDs can be formulated to selectively release siRNA in conditions where temperature, light, pH, and redox microenvironment [16] are modified. Recent advances in CD-based supramolecular systems have underscored the central role of both thermodynamic stability and kinetic accessibility in the design of effective gene-delivery platforms. The review of Zhang et al. (2020) highlights how host–guest interactions, threading or sliding of CD rings, and stimuli-responsive triggers (pH, redox, enzyme, and light) enable rapid morphological and functional transitions of CD nanocarriers, thereby influencing both the rate of assembly/disassembly and the release kinetics of the cargo under physiological conditions [64]. In parallel, the review of Mousazadeh H. et al. (2021) discusses how CD-based carbohydrate polymers (including CD-cationic polymers, CD-polyrotaxanes, CD-dendrimers, and CD-modified targeting ligands) can be finely tuned in terms of degree of functionalization, N/P ratio (that represents the molar ratio between the nitrogen atoms in the cationic components and the phosphate groups of the siRNA backbone [65]), ionic microenvironment, and presence of co-polymers to modulate both binding affinity and complex formation/release behavior of siRNA delivery systems [16]. Overall, these works suggest that the thermodynamics and kinetics of the self-assembly process in CD-based siRNA delivery systems are critically governed by multiple physicochemical parameters, including the CD substitution pattern and degree of functionalization, the ionic strength and pH of the surrounding medium, the N/P ratio (nitrogen in CD to phosphate in siRNA), and the presence of co-polymers or stabilizers such as PEG, hyaluronic acid, or chitosan. Isothermal titration calorimetry (ITC) has emerged as a powerful technique to quantify the binding thermodynamics of CD–siRNA interactions, providing access to parameters such as binding affinity (K_d), enthalpy (ΔH), entropy (ΔS), and Gibbs free energy (ΔG) [50,66]. Studies on self-assembled cationic β-CD nanostructures have demonstrated that the substitution degree of cationic groups markedly influences the electrostatic contribution to ΔH and ΔS, with higher substitution leading to stronger exothermic interactions and reduced entropic penalties during complexation [5]. In particular, the entropic loss in the complexation of self-assembled cationic β-CDs mainly arises from a reduced conformational freedom of both CDs and guest molecules, as well as from the ordering of water molecules and counterions around the complexes. The enthalpic contribution is due to β-CDs stronger binding (ΔH < 0) to negatively charged molecules like siRNA when cationic groups are present in their structures. In this latter case, however, the entropic penalty is reduced because more water molecules and counterions are released during binding, which partially compensates for the loss of flexibility.

Recent molecular dynamics simulations and free energy studies have emphasized the critical role of solvent reorganization in the entropic term, showing that the release or rearrangement of water molecules and ions upon complex formation significantly contributes to ΔS and thus to the overall binding stability [67,68,69].

The ionic strength and pH of the medium are able to further modulate complex stability by altering electrostatic screening and protonation states, shifting the balance between enthalpic and entropic driving forces [7]. From a kinetic perspective, the dynamics of siRNA complexation and release strongly depends on the N/P ratio and the nature of co-stabilizing polymers. Elevating N/P ratios typically enhances complex compactness and reduces dissociation rates, whereas the inclusion of PEG, hyaluronic acid, or chitosan can modulate assembly kinetics and colloidal stability by steric or electrostatic contributions [70,71]. ITC combined with molecular dynamics simulations has revealed that self-assembled β-CD/siRNA complexes exhibit favorable binding kinetics driven primarily by electrostatic and dehydration effects [66]. Moreover, differential scanning calorimetry (DSC) and fast-scanning calorimetry (FSC) have been used to probe solid-state transitions and thermal stability, confirming that the substitution pattern and N/P ratio affect not only molecular affinity but also phase behavior and thermal robustness [7,72].

Overall, the interplay between thermodynamic stability (ΔG, ΔH, ΔS), solvent reorganization, and kinetic accessibility dictates the efficiency of siRNA complex formation, protection, and intracellular release.

## 4. Computational-Experimental Design of β-Self-Assembling CD

Singh et al. (2019) describe the design and characterization of self-assembled cationic β-CD (cCD) nanostructures for siRNA delivery, employing a combined computational and experimental approach [66]. Using extensive molecular dynamics simulations, they demonstrate that cCD molecules spontaneously assemble into supramolecular bilayer-like structures around siRNA, stabilizing its native conformation via extensive electrostatic interactions and hydrogen bonding. Unlike unmodified β-CDs, which form transient, non-specific complexes, cCD derivatives exhibit strong, specific binding to siRNA, mediated by their positively charged side arms and hydrophobic alkyl chains. These simulations reveal lipid-like interdigitated assemblies that encapsulate siRNA, mimicking natural biomembranes and potentially enhancing membrane permeability. Isothermal titration calorimetry experiments validate all these findings, confirming a spontaneous, enthalpy-driven complexation process with low dissociation constants.

## 5. Modified CD for Receptor-Mediated Targeted Delivery

Targeted formulations can achieve superior knockdown efficiency compared to non-targeted controls. Examples of this important issue have been described above in Section Stimuli Responsive and Thermodynamics in CD-Mediated Gene Delivery, since stimuli-responsive vectors are a first class of targeted systems. More specifically, CD can be modified to allow receptor-mediated delivery, particularly useful for cancer therapy. Malhotra et al., 2018, prepared ligand-targeted nanoparticles by exploiting the inclusion complexation capabilities of CD and adamantyl-PEG-modified ligands combined with chitosan, obtaining a good internalization to glioblastoma (U87) and prostate cancer (DU145) cells [51]. This performance has been attributed to the formation of supramolecular structures through interdigitation of aliphatic tails for disulfide-linked conjugates, which demonstrated enhanced gene silencing, likely due to intracellular bioreduction [73]. Li et al. 2023 developed a folic acid-functionalized β-CD-grafted polyethylenimine (β-CD-PEI-FA) nanocarrier for the targeted delivery of miR-34a-5p against Kaposi’s sarcoma-associated herpesvirus (KSHV) [74]. The β-CD-PEI-FA polymer formed stable nanocomplexes with miR-34a-5p via electrostatic interaction, effectively protecting the miRNA from nuclease and serum degradation. The nanocomplex exhibited suitable physicochemical properties (size ~203 nm, zeta potential ~27 mV) for cellular uptake and showed low cytotoxicity and hemolysis in vitro. Functional assays in KSHV-positive BCBL-1 and SK-RG cells demonstrated that β-CD-PEI-FA/miR-34a-5p complexes increased intracellular miR-34a-5p levels, inhibited cell proliferation by arresting cells in the G2 phase, and significantly downregulated KSHV genes (ORF26, LANA, and K8.1A). These results suggest that β-CD-PEI-FA represents a promising strategy for folate-receptor-targeted delivery of therapeutic miRNAs in antiviral applications. Table 3 provides an expanded qualitative comparison of cyclodextrin derivatives, functionalization strategies, and biological outcomes, together with the corresponding level of experimental validation.

Most of the CD-based nanovectors reported to date have been primarily evaluated at the in vitro level, demonstrating efficient siRNA complexation, cellular uptake, and gene silencing in cancer and neuronal cell models, while in vivo validation remains limited to selected systems.

## 6. Translational Barriers

One of the principal challenges associated with cationic cyclodextrin derivatives is their complex and costly synthesis. These systems are generally produced on a small scale and often involve the use of environmentally hazardous reagents and organic solvents. Moreover, purification procedures frequently rely on techniques such as chromatographic separation, which are not easily scalable or economically sustainable for industrial production. Consequently, the development of green and scalable approaches for both synthesis and purification remains highly challenging [32,75].

Another important limitation concerns the selection of the most appropriate siRNA sequence for specific clinical applications. Careful patient stratification and target validation are required, which may delay patient enrollment in Phase I clinical trials; however, this strategy can significantly enhance the likelihood of accurately evaluating the therapeutic efficacy of siRNA in controlling the intended molecular target [16].

Furthermore, to improve selectivity and reduce potential off-target effects, nanovectors are often functionalized with targeting ligands capable of recognizing specific disease-associated cells or tissues. The field of targeted delivery has advanced rapidly in recent years, with significant progress in understanding biological barriers and in developing innovative strategies to functionalize nanocarriers with targeting moieties for accurate and selective delivery. Nevertheless, increasing structural complexity, such as the incorporation of multiple functional components, further complicates large-scale industrial production. Despite these challenges, emerging strategies, including multifunctional nanovector design and machine learning-assisted optimization, are expected to facilitate the development of safer, cell-specific, and more scalable nanoparticle delivery systems.

## 7. Conclusions

CD-based NVs are versatile and promising platforms for siRNA delivery, thanks to their structural adaptability, biocompatibility, and tunable host–guest interactions. The ability to modulate the physicochemical parameters of CDs, such as degree of substitution, cationic charge density, and functionalization with targeting or stimuli-responsive groups, allows fine control over complex stability, binding thermodynamics, and release kinetics under physiological conditions. Modified CD-based nanocarriers are capable of effectively condensing and protecting siRNA, facilitating its cellular uptake while minimizing cytotoxicity and immune responses. Self-assembled CD-based nanovectors rely on supramolecular host–guest interactions, offering greater formulation flexibility, adaptability, and translational potential.

Overall, the effectiveness of cyclodextrin-based nanovectors for siRNA delivery is strongly governed by structure–function relationships, since subtle variations in molecular architecture and supramolecular organization markedly influence biological performance. Key parameters such as degree of substitution, cationic charge density, nanovector architecture, and surface functionalization determine siRNA condensation, complex stability, intracellular trafficking, and cytotoxicity. Optimizing these features is essential to balance efficient cellular uptake and endosomal escape with serum stability and biocompatibility, underscoring the need for rational design strategies that integrate molecular structure, supramolecular dynamics, and biological response.

As yet, despite the significant progress reported, most CD-based siRNA systems remain supported predominantly by in vitro evidence, with a limited number validated in vivo and only a few reaching clinical trials. This highlight both the promise of cyclodextrin-based platforms and the need for further translational studies addressing biodistribution, long-term safety, and therapeutic efficacy.

## 8. Future Perspectives

Looking forward, a particularly promising direction is the development of multi-responsive CD architectures tailored to the tumor microenvironment (TME). In TME, conditions such as mildly acidic extracellular pH, low endosomal/lysosomal pH after uptake, and elevated intracellular reducing power represent exploitable triggers for controlled release [76,77]. By designing CD-based nanocarriers that respond to both pH and redox stimuli, it should be possible to achieve highly selective and efficient intracellular siRNA release upon tumor cell internalization. Dual pH/redox-responsive systems have already shown promise in non-CD nanocarriers for drug or siRNA delivery [78,79]. Moreover, incorporating targeting ligands (e.g., tumor-targeting peptides, hyaluronic acid, and antibodies) onto CD could enhance specificity and reduce off-target uptake.

To facilitate eventual clinical translation, future efforts should also focus on biodegradable and scalable CD-based polymers, as well as on detailed mechanistic studies to better understand how molecular structure, supramolecular dynamics, and environmental triggers (pH, redox, and ionic strength) influence siRNA complexation, release kinetics, intracellular trafficking, and gene silencing efficiency. The integration of dual-stimuli responsiveness, targeting functionality, and biocompatibility may offer a powerful route toward safe, efficient, and precise systemic siRNA delivery.

Furthermore, as reported for other nanovectors, the impact of carrier shape and size, surface charge, polydispersity index, and related physicochemical features on biocompatibility should be systematically investigated. In particular, long-term in vivo studies remain limited, hampering the translation of laboratory findings into clinical applications. Careful monitoring of renal and hepatic functions, as well as assessment of cumulative toxicity risk, will be essential before clinical implementation [80].

Off-target effects and non-specific gene silencing remain important limitations of siRNA therapeutics. These issues can be mitigated by optimizing siRNA size, primary sequence, and targeting location. The ideal nanovector should have a size between 50 and 200 nm to achieve effective delivery to target cells while minimizing off-target effects, increasing resistance to nuclease degradation, and preventing immune responses [81]. In this context, advanced protein array technologies are expected to play an increasingly relevant role, as they provide a more comprehensive picture of siRNA effects on the cellular protein expression profile. This approach is increasingly applied to evaluate protein interactions, expression profiling, and target discovery, helping to resolve undesired targeting. Protein microarrays are versatile tools for parallel, miniaturized screening of binding events involving large numbers of immobilized proteins in a time- and cost-effective manner [16,82].

In parallel, in silico studies are increasingly used for pre-development predictions to understand the nature and structure of active compounds, their interactions, and to identify potential toxicities through computational models such as molecular dynamics simulations, docking, and Quantitative Structure–Activity Relationship (QSAR) approaches. These methodologies are also employed to investigate complexation properties and to model NP–membrane interactions and cellular uptake based on minimal physicochemical data. More importantly, machine learning enables computational models to predict how specific modifications in nanovector physicochemical characteristics may improve functional aspects such as drug retention and endocytosis efficiency. On a larger scale, these tools may also predict in vivo pharmacokinetics of the encapsulated drugs, including circulation half-life, toxicity, and biodistribution. However, the convergence of nanomedicine and computational approaches is still in its early stages and requires further validation to achieve reliable translational impact [83,84].

## Figures and Tables

**Table 1:** Summary of the main non-viral nanovectors discussed in this review, highlighting advantages and limitations.

NVs Type	Advantages	Limitations	References
CD NVs	High structural tunability, good siRNA complexation, high stability, promising transfection, and silencing results.	Scalable manufacturing and in vivo biodistribution data are less mature; endosomal escape can be challenging.	[13,15,16,31,32,49,50]
LNPs	Clinically validated; high encapsulation efficiency; excellent endosomal release; scalable GMP manufacturing.	Can induce immune activation; PEGylation can cause accelerated blood clearance and immune issues; targeting beyond the liver remains challenging.	[20,21,23]
Polymeric NVs	Large chemical/design space, tunable release, and mechanical stability; good protection of siRNA.	Potential cytotoxicity (especially non-degradable cationic polymers); heterogeneous formulations; less clinical precedence than LNPs.	[26,27]
siRNA conjugates	Highly defined stoichiometry; excellent potency for accessible tissues; simplified formulation (no nanoparticle vehicle needed).	Tissue scope limited by target receptor expression and by endosomal escape; designing stable conjugates for non-hepatic targets is challenging.	[28,29]

**Table 2:** Review of CDs for gene delivery cited in the literature.

Authors	Year	Title	References
Chaturvedi, K.	2011	Cyclodextrin-Based siRNA Delivery Nanocarriers: A State-of-the-Art Review.	[15]
Xu, C.	2019	Cyclodextrin-Based Sustained Gene Release Systems: A Supramolecular Solution towards Clinical Applications.	[13]
Haley, R.M.	2020	Cyclodextrins in Drug Delivery: Applications in Gene and Combination Therapy.	[49]
Mousazadeh, H.	2021	Cyclodextrin-Based Natural Nanostructured Carbohydrate Polymers as Effective Non-Viral siRNA Delivery Systems for Cancer Gene Therapy.	[16]
Castillo Cruz, B.	2022	A Fresh Look at the Potential of Cyclodextrins for Improving the Delivery of siRNA Encapsulated in Liposome Nanocarriers	[50]
Nazli, A.	2025	Cationic Cyclodextrin-Based Carriers for Drug and Nucleic Acid Delivery	[32]

**Table 3:** CD nanovectors for gene delivery from 2020 to 2025.

CD Derivative/Modification	Functionalization Strategy	siRNA/Target Gene	Biological Outcome	Level of Validation	References
TEPA-β-CD polyplexes	Cationic polymer functionalization	anti-GFP	Efficient siRNA complexation and gene silencing with low cytotoxicity	In vitro	[71]
β-CD derivatives/β-CD–Ad–PEG/anisamide ligand	Amphiphilic CD + PEGylation + targeting ligand	PLK1	Enhanced cellular uptake and receptor-mediated targeting in cancer cells	In vitro	[31]
Surface-modified β-CDs with RVG peptide	Cationic CD + peptide targeting	HTT mRNA	Effective BBB crossing and neuronal gene silencing	In vitro	[53]
Modified cationic β-CD–Ad–PVA–PEG	Host–guest self-assembly + cationic CD	anti-GFP	High siRNA loading, efficient silencing, low cytotoxicity	In vitro	[56]
Amphiphilic cationic CD/anionic CD co-formulation	Charge-balanced amphiphilic CDs	KAT2a	Reduced surface charge, preserved uptake, and effective gene knockdown	In vitro	[57]
Sialic-acid-functionalized CD nanoparticles	Targeting ligand (Siglec-1)	CSF-1R	Selective uptake by TAMs and macrophage repolarization	In vitro	[58]
AS1411 aptamer–PD-L1 siRNA + Gln–CM–β-CD/PEI–DOX	Targeting aptamer + CD–polymer hybrid	PD-L1	Tumor growth inhibition, immune activation, reduced toxicity	In vivo	[59]
CD–polymer–PEG with transferrin ligand (CALAA-01)	Targeted CD polymeric NV	RRM2	First evidence of RNAi-mediated gene silencing in humans	Clinical trial	[60]
Modified cationic β-CD nanostructures	Self-assembled cationic CD	PLK1	Stable complexation, efficient intracellular delivery	In vitro	[66]
Covalent β-CD–siRNA conjugates	Covalent conjugation	PLK1/GFP	Preserved silencing activity with improved stability	In vitro	[51]
FA–β-CD–PEI copolymer	Targeting ligand (folic acid) + cationic polymer	miR-34a-5p	Receptor-mediated uptake and antiviral gene regulation	In vitro	[70]

## Data Availability

No new data were created or analyzed in this study. Data sharing is not applicable to this article.

## Abbreviations

The following abbreviations are used in this manuscript:

siRNA	small interfering RNA
CD	cyclodextrin
NVs	nanovectors
RNAi	RNA interference
miRNA	microRNA
LNPs	lipid nanoparticles
hATTR	hereditary transthyretin amyloidosis
BBB	blood–brain barrier
cCD	cationic cyclodextrin
AML	acute myeloid leukemia
TAMs	tumor-associated macrophages
